# An interpretable approach for social network formation among heterogeneous agents

**DOI:** 10.1038/s41467-018-07089-x

**Published:** 2018-11-08

**Authors:** Yuan Yuan, Ahmad Alabdulkareem, Alex ‘Sandy’ Pentland

**Affiliations:** 1 Institute for Data, Systems, and Society, Massachusetts Institute of Technology, Cambridge, MA 02139 USA; 2 Center for Complex Engineering Systems, King Abdulaziz City for Science and Technology and Massachusetts Institute of Technology, Riyadh, 12354 Saudi Arabia; 3 Media Lab, Massachusetts Institute of Technology, Cambridge, MA 02139 USA

## Abstract

Understanding the mechanisms of network formation is central in social network analysis. Network formation has been studied in many research fields with their different focuses; for example, network embedding algorithms in machine learning literature consider broad heterogeneity among agents while the social sciences emphasize the interpretability of link formation mechanisms. Here we propose a social network formation model that integrates methods in multiple disciplines and retain both heterogeneity and interpretability. We represent each agent by an “endowment vector” that encapsulates their features and use game-theoretical methods to model the utility of link formation. After applying machine learning methods, we further analyze our model by examining micro- and macro- level properties of social networks as most agent-based models do. Our work contributes to the literature on network formation by combining the methods in game theory, agent-based modeling, machine learning, and computational sociology.

## Abstract (web summary)

Complex networks can be a useful tool to investigate problems in social science. Here the authors use game theory to establish a network model and then use a machine learning approach to characterize the role of nodes within a social network.

## Introduction

Social networks have attracted increasing attention from both physical and social scientists^1–4^. Social networks are essential elements in societies, serving as channels for exchanging various benefits, such as innovation, information, and social support^5–8^. Moreover, research in social networks helps explain macro-level social phenomena, such as social polarization^9^ and social contagion^10,11^. An understanding of social networks has significant implications, such as improving social welfare and political participation^12,13^.

Previous work on modeling social network formation has typically employed game theory or agent-based modeling^14–20^. These studies typically propose simple and tractable micro-level rules for link formation mechanisms and show that these rules have implications for known macro-level properties. Several studies in statistics and econometrics have also used game theory to model empirical networks^21–23^, but they typically have been focused on estimating and identifying the effects of interest, such as racial segregation. To date, these models have not been capable of accounting for the effects of broad heterogeneity among individuals; therefore, they lack predictive power for link formation in complex, real-world networks.

Studies on network embedding techniques^24–27^ could partially fill this gap in the network formation literature because these techniques consider node heterogeneity and show predictability of both link formation and individual characteristics. Network embedding techniques are aimed at representing each node with a fixed-length vector learned from social network data. The agents in a network may be so diverse that representing all their characteristics would require very high dimensionality for these vectors. The philosophy of network embedding is aimed at reducing the dimensionality by mapping all the characteristics of agents onto a low-dimensional latent space. Each dimension in the latent space, therefore, typically does not correspond to a concrete attribute of the agents. The latent space representation of nodes on a network provides considerable potential for measuring heterogeneity among agents. However, because network embedding methods are designed for data representation and compression rather than for explaining network formation, they do not attempt to capture micro, inter-agent effects such as social status or macro effects such as social segregation; thus, they do not provide social science explanations for the link formation.

There are few network formation papers that have attempted to account for heterogeneity of agent without losing micro-level interpretability. A study on ecological networks by McKane and Drossel utilized a similar approach, wherein agents are represented by a small number of attributes among a large attribute pool^28^. However, this work does not directly estimate the latent variables for networks of agents. More abstractly, our method is also reminiscent of mixed membership stochastic blockmodels where agents respectively follow a probability distribution of membership within several communities^29^. However, probabilistic membership models typically do not seek to uncover economic and sociological mechanisms and the dynamics of network formation. We extend these previous works to the estimation of agent characteristics and network link formation using observed network data. In addition, we want to incorporate a more complex but interpretable inter-agent exchange utility function, by modeling both exchange benefits and coordination costs arising from the differences among agents.

Furthermore, an important question rarely studied in literature is the trade-off between coordination costs and exchange benefits. On the one hand, the coordination between two dissimilar agents incurs higher coordination costs than between two similar agents^30^, a relationship which encourages homophily, i.e., the tendency to interact more with agents who have shared characteristics^31^. On the other hand, the rationale of exchange benefits comes from welfare economics: agents have different endowments and their preferences drive different agents to interact and exchange endowments^32^. The exchange nature therefore encourages heterophily, i.e., the tendency to interact with dissimilar individuals^33^. Empirical studies have found that heterophily exists in various scenarios^34,35^, and that complimentary heterophily between two agents sometimes bring more mutual benefits than homophily^36^. However, most prior studies of social network formation consider either only coordination costs and homophily^22,37,38^ or only social exchange benefits and heterophily^39–41^, rather than an integration of exchange and coordination as we do in this paper. The trade-off between exchange benefits and coordination costs is also reminiscent of the identity-diversity balance in the organizational performance literature^42,43^.

In this paper, inspired by the network embedding techniques, we develop a social network formation model using representation learning methods for heterogeneous agents; to retain the interpretability, we maintain the inter-agent micro-structure characteristics of most agent-based models and the macro-level structures that are the focus of sociology. In our model, agents are characterized by vectors, called their endowment vectors; agents maximize their utility by having link formation driven by comparing their own endowment vectors with those of others. Importantly, we take an economic view of human networks, which considers link formation to be driven by the trade-off between the benefit of exchanges^44^ among individuals with different endowments against the coordination costs due to differences in some other dimensions of endowments. We apply optimization methods to ascertain the endowment vectors of all agents from empirical social networks. The effectiveness of this method is validated by prediction tasks of link formation and individual characteristics. Subsequently, the agent-based models derived from empirical data are evaluated in terms of their micro- and macro-level behavior, compared with the behavior of human networks. Abstractly, we model link formation as a reaction-diffusion system, a framework found in many biological systems.

## Results

### A game theoretical model

Endowment is a well-known and useful concept in microeconomic theory^32^, for example, fundamental theorems of welfare economics are based on agent exchanging endowments. In our model, an endowment vector could potentially represent all of the features (assets, abilities, capacities, qualities, etc.) that each agent possesses, and are treated as fixed, invariant characteristics of the agent. We do not consider the situation where endowments are dynamic in this study. Since we limit the dimensionality of endowment vectors, similar to network embedding algorithms (see Methods), each dimension does not necessarily have a specific meaning, but may be a combination of many attributes of an individual.

Agents establish social ties according to the comparison between their endowments. If we assume that there are *K* dimensions of endowments in a society, each agent has a *K*-dimensional endowment vector **w**. Note that dimensions may be mutually correlated; for example, in the Karate club network, leaders and followers have high values in their respective dimensions, and these two dimensions should be negatively correlated. We constrain the first and second moments of each dimension $\left( { {{\mathbf{W}}_{:k}}} \right)$ to be zero and one, respectively, for computational simplicity.

We assume the utility function of agent *i* is only determined by agent *i*’s neighbors’ endowment vectors. We define the utility function $U_i:2^{{\cal I}/\{ i\} } \to {\Bbb R}$ for all *i*, as Eq. (1). The argument *S* is the potential neighbors, denoting an arbitrary subset of all agents except *i* herself, i.e., ${\cal I}\{ i\}$. Each agent *i* selects her neighbor set *S* by maximizing her utility function *U*_*i*_. *U*_*i*_ is composed of two terms, the benefits of exchange (*F*_*i*_) and the costs of coordination (*G*_*i*_):

1 $$U_i(S;{\mathbf{W}},{\mathbf{b}},{\mathbf{c}}) = \underbrace {F_i(S;{\mathbf{W}},{\mathbf{b}})}_{{\mathrm{benefits}}\,{\mathrm{of}}\,{\mathrm{exchange}}} - \underbrace {G_i(S;{\mathbf{W}},{\mathbf{c}})}_{{\mathrm{costs}}\,{\mathrm{of}}\,{\mathrm{coordination}}},\quad \forall S \subset {\cal I}\{ i\} .$$

Let $S_i^ \ast$ be the optimal neighbor set for *i*. We define the marginal utility that *j* brings to *i* as:

2 $${\mathrm{\Delta }}u_i(j) = \left\{ {\begin{array}{*{20}{l}} {U_i(S_i^ \ast ;{\mathbf{W}},{\mathbf{b}}) - U_i(S_i^ \ast /\{ j\} ;{\mathbf{W}},{\mathbf{b}}),} \hfill & {{\mathrm{if}}} \hfill & {j \hskip 4pt \in \hskip 4pt S_i^ \ast ;} \hfill \\ {U_i(S_i^ \ast \cup \{ j\} ;{\mathbf{W}},{\mathbf{b}}) - U_i(S_i^ \ast ;{\mathbf{W}},{\mathbf{b}}),} \hfill & {{\mathrm{if}}} \hfill & {j \hskip 4pt\notin \hskip 4pt S_i^ \ast .} \hfill \end{array}} \right.$$

In this study, we are focused on specific forms for *F*_*i*_ and *G*_*i*_ and, consequently, for *U*_*i*_. For the costs of coordination, agent *i*’s cost incurred by agent *j* is measured by the difference between **w**_**j**_ and **w**_**i**_.

3 $$G_i(S;{\mathbf{W}},{\mathbf{c}}) = \mathop {\sum}\limits_{i \in S} g({\mathbf{w}}_{\mathbf{j}},{\mathbf{w}}_{\mathbf{i}},{\mathbf{c}}) = \mathop {\sum}\limits_{i \in S} \left\| {{\mathbf{c}} \circ ({\mathbf{w}}_{\mathbf{j}} - {\mathbf{w}}_{\mathbf{i}})} \right\|_2.$$

“$\circ$” denotes element-wise multiplication. $\left\| x \right\|_2$ denotes $\ell _2$ norm. Note that the costs are symmetric, i.e., $\left\| {{\mathbf{c}} \circ ({\mathbf{w}}_{\mathbf{i}} - {\mathbf{w}}_{\mathbf{j}})} \right\|_2 = \left\| {{\mathbf{c}} \circ ({\mathbf{w}}_{\mathbf{j}} - {\mathbf{w}}_{\mathbf{i}})} \right\|_2$. The costly scaling parameter, *c*_*k*_, measures the importance of *k*-th dimensions on the costs. A higher *c*_*k*_ will amplify the difference between *i* and *j*’s endowment vectors on the *k*-th dimension (*w*_*jk*_–*w*_*ik*_). This term encourages homophily: dissimilar pairs have to suffer from high coordination costs before forming a link.

For *F*_*i*_, we propose the following form:

4 $$F_i(S_i^ \ast ;{\mathbf{W}},{\mathbf{b}}) = \mathop {\sum}\limits_{j \in S_i^ \ast } \mathop {\sum}\limits_{k = 1}^K b_k\,{\mathrm{max}}(w_{jk} - w_{ik},0).$$

Intuitively, *w*_*jk*_–*w*_*ik*_ measures the “advantage” of agent *j* on the *k*-th dimension over agent *i*. As we do not want negative benefits, we consider the benefit on the *k*-th dimension is zero if *w*_*jk*_–*w*_*ik*_ < 0. In deep learning, max(*x*, 0) is called the “ReLU” function. TensorFlow^45^, a machine learning programming library, provides methods to optimize functions that contain ReLU functions. Similar to *c*_*k*_, the beneficial scaling parameter *b*_*k*_ measures how beneficial the *k*-th dimension is. This term indicates that when an agent is high in several dimensions, she could bring high benefits to others. Therefore, other agents are inclined to link to her. However, she does not necessarily reciprocate every link because, for example, when she is higher in every dimension than others, she will not benefit from others in any dimension. Note that for simplicity, we do not consider comparative advantages in this paper. In addition, this term encourages heterophily: agents whose expertises are complimentary have high potential benefits for link formation. Therefore, in this specific form, we have

5 $${\mathrm{\Delta }}u_i(j) = \mathop {\sum}\limits_{k = 1}^K b_k{\mathrm{max}}(w_{jk} - w_{ik},0) - \left\| {{\mathbf{c}} \circ ({\mathbf{w}}_{\mathbf{j}} - {\mathbf{w}}_{\mathbf{i}})} \right\|_2$$

There are of course many other variations for the functional form (Eq. (1)). For example, we can let *F*_*i*_ non-separable in terms of the neighbor set *S*, e.g., $F_i(S) = \frac{1}{{|S|}}\mathop {\sum}\nolimits_{j \in S_i^ \ast } \mathop {\sum}\nolimits_{k = 1}^K b_k\,{\mathrm{max}}(w_{jk} - w_{ik},0)$. The intuition is that when one agent has many neighbors, the benefit brought by each neighbor decreases; Do et al. provide a good example of a decreasing marginal utility^46^. However, this functional form indicates that Δ*u*_*i*_(*j*) depends on the neighbor set *S*, which leads to a time-consuming combinatorial optimization in the learning process; specifically, when the learning algorithm chooses $S_i^ \ast$, it may need ${\cal O}(N2^N)$ computations for the utility functions, which is computationally infeasible for even a small-scale network. This is thus beyond the scope of this paper. We can also change *G*_*i*_ into other norms, such as $\ell _1$ norm, or change *F*_*i*_ into a smoother version of max(*x*, 0), but these changes do not significantly affect the results in the later sections, as shown in Supplementary Note 9. Therefore, we concentrate on this specific form in later sections (Eq. (5)).

In network game theory, pairwise stability^20^ refers to the situation where no increased marginal utility can be brought to both agents of an unconnected pair, and no increased marginal utility can be brought to any agents who want to drop their neighbors. Following the definition, we derive the conditions when pairwise stability in undirected networks is satisfied. The proof is straightforward and can be found in Supplementary Note 1.

### Proposition 1

An undirected network $\left( {{\cal G} = ({\cal V},{\cal E})} \right)$ implied by neighbor sets $S_i^ \ast$, *i* = 1,2,...,*N* is pairwise stable, if the following conditions are satisfied:

1. if $$j \in S_i^ \ast$$, then $$i \in S_j^ \ast$$;
2. $$\forall j \in S_i^ \ast$$, Δ*u*_*i*_(*j*) ≥ 0;
3. $$\forall j \notin S_i^ \ast$$, min(Δ*u*_*i*_(*j*), Δ*u*_*j*_(*i*)) < 0.

### Learning endowments

We have established a model for social network formation with many parameters and latent variables. Before we examine the proprieties of the model, we have to assign values for the unknown variables, including the endowment vectors (**W**), and scaling parameters (**b** and **c**). To equip our model with the capability of fitting real-world networks, we learn the endowment vectors using the observations of real-world networks, by assuming real-world networks are at or close to pairwise stability.

Let ${\cal L}({\mathbf{b}},{\mathbf{c}},{\mathbf{W}}|D)$ be the loss function that we want to minimize. The definition of ${\cal L}({\mathbf{b}},{\mathbf{c}},{\mathbf{W}}|D)$ is reported in Supplementary Note 3. Then we solve the optimization problem in Eq. (6).

6 $$\begin{array}{*{20}{l}} {{\mathrm{Minimize}}_{{\mathbf{b}},{\mathbf{c}},{\mathbf{W}}}:} \hfill & {{\cal L}({\mathbf{b}},{\mathbf{c}},{\mathbf{W}}|D)} \hfill \\ {{\mathrm{Subject}}\,{\mathrm{to}}:} \hfill & {b_k \ge 0{\mathrm{,}}\forall k = 1,2,...K} \hfill \\ {} \hfill & {c_k \ge 0{\mathrm{,}}\forall k = 1,2,...K} \hfill \\ {} \hfill & {\frac{{\mathop {\sum}\limits_{i = 1}^N w_{ik}}}{N} = 0{\mathrm{,}}\forall k = 1,2,...K} \hfill \\ {} \hfill & {||{\mathbf{W}}_{\mathbf{: k}}||_2^2 = N{\mathrm{,}}\forall k = 1,2,...K} \hfill \end{array}$$

The constraints that *b*_*k*_ and *c*_*k*_ should not be less than 0 are required by the properties of our model. The constraint for the mean of each dimension is to limit the number of equivalent solutions, so that the optimizer could typically find a better solution. The constraint of **W**_**:**__**k**_ is to guarantee that the standard deviation of each dimension is approximately 1, so that the values of **b** and **c** are comparable across dimensions.

As ${\cal L}({\mathbf{b}},{\mathbf{c}},{\mathbf{W}}|D)$ is nonlinear and non-convex (dimensions are interchangeable) with respect to (**b**, **c**,**W**), we have to approximate the global optimum by a local optimum. By employing Adam optimizer (an improved stochastic gradient descent method)^47^, we are able to learn the local optimum of ${\cal L}({\mathbf{b}},{\mathbf{c}},{\mathbf{W}}|D)$; Adam optimizer is good at deriving satisfying local optima when solving nonlinear and non-convex problems. To obtain a solution that approximates the global optimum, we start from many randomly selected initial points and then analyze the results of the multiple runs to find the parameters that generate the smallest loss and therefore the best link fitting performance. Technical details, including the definition of ${\cal L}$ and methods that assist learning, are presented in Supplementary Note 3.

### Validation of learning

Here we show that we have learned meaningful endowment vectors from empirical networks. In particular, we first use a toy example—Zachary’s karate club network^48^ to illustrate the learned results. We then validate the effectiveness of our model and learning method by showing their performance at fitting link formation and predicting individual characteristics for a variety of large-scale social networks: a synthetic network where two types of agents exchange, a Trade network among countries, a movie collaboration network, a Company communication network, and the Andorra network, which is a nationwide mobile phone network (see Methods).

**Fig. 1:**
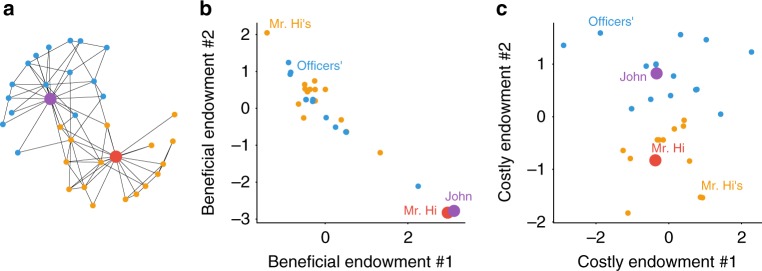
Illustration of the learned endowments for karate club network. **a** The network structure of karate club network. Mr. Hi, the instructor, is marked red, and other people in his faction are marked orange. John, the student leader or officer, is marked purple, and other people in his faction are marked blue. **b** The first two dimensions of the learned endowment vector for each individual; these two dimensions are only related to exchange benefits so we call them “beneficial endowments”. **c** The last two dimensions of the learned endowment vector for each individual; these two dimensions are only related to coordination costs so we call them “costly endowments”

We start with a toy example to illustrate both the rationale of the present model and the effectiveness of learning performance. Because of a conflict between an instructor (Mr. Hi) and a student officier (John), the social network of Zachary’s karate club is polarized into two factions (Fig. 1a). We set *K* = 4 and the first two dimensions as “beneficial endowments” and the last two dimensions “costly endowments” (Methods section) because it is more convenient for visualization if the numbers of beneficial and costly dimensions are both even. Note that *K* = 4 is not necessarily the optimal dimensionality and here we did not add a regularization term (Supplementary Note 3) for this result; however, we also show in Supplementary Note 8 that *K* = 4 is a reasonable (almost optimal) selection.

Panels b and c in Fig. 1 plot the values of the learned endowments of individuals in Zachary’s karate club. In panel b, both Mr. Hi and John are high in dimension #1 and low in dimension #2, while the rest are generally low in endowment dimension #1 and high in dimension #2. We interpret this result as the tendency of exchanges between instructors and students: dimension #1 represents the professional skill of karate and leadership in their factions; endowment #2 represents the willingness to learn Karate. As for costly endowments (panel c), we find that dimension #4 corresponds to the faction to which each individual belongs: Mr. Hi and his followers (orange) have values generally higher than 0 while John and his followers (blue) are generally lower than 0. Dimension #4 can be explained as the individual’s identification with the two factions. We interpret cost endowment #3 as other unobserved characteristics that might influence the interactions between individuals, such as the time and frequency to participate in club activities. We also illustrate the learning results for the Trade and Synthetic datasets graphically in Supplementary Note 4.

Because our goal is to use the learned endowment vectors to further analyze the micro- and macro- patterns of the network, we learn the endowment vectors by using all the information (the links) of the network. Therefore, rather than split the input links into training and test sets, we use all the links as the input. A potential concern is that we might “overfit” the network by using a large *K*; we partially address this concern by introducing the regularization term ${\cal L}_{{\mathrm{reg}}}$ as mentioned in Supplementary Note 3. We use Δ*u*_*i*_(*j*) as the predictor and AUC (area under the curve) as the measurement for the fitting performance. AUC trades off between true positive and false positive rates, and serves as a fair measure when there is a strong imbalance between positive and negative samples. By using an approach provided in Supplementary Note 3, we obtain the optimal dimensionality (*K*) and the optimal number of beneficial and costly endowments (*K*_bnf_ and *K*_cst_, see their definitions in Methods).

As shown in Table 1, our model is able to obtain very good fits to the input networks. For all datasets, the AUC of link fitting is over 94%. Moreover, we demonstrate that for all datasets, it is necessary to incorporate both the benefit and the cost terms into the utility functions (i.e., *K*_bnf_ > 0 and *K*_cst_ > 0). This finding highlights the importance of integrating both exchange effects and coordination costs into the link formation mechanisms. Other technical details, including learning curves and the performance on all the dimensions, are presented in Supplementary Note 3.

**Table 1:** Learning results and link fitting performance of learned endowment vectors

Dataset	$|{\cal V}|$	$|{\cal E}|$	*K* ^*^	$K_{{\mathrm{bnf}}}^ \ast$	$K_{{\mathrm{cst}}}^ \ast$	AUC
Karate	34	78	3	2	1	98.48%
Trade	100	703	5	3	2	96.85%
Synthetic	2500	14,453	4	2	2	99.92%
Company	1984	12,751	11	4	7	98.70%
Movie	2788	10,399	7	4	3	96.08%
Andorra	32,829	513,931	15	8	7	94.76%

$|{\cal V}|$ denotes the number of nodes and $|{\cal E}|$ denotes the number of edges. K^*^, $K_{{\mathrm{bnf}}}^ \ast$, and $K_{{\mathrm{cst}}}^ \ast$ represent the optimal number of dimensions, beneficial dimensions, and costly dimensions, respectively

Although our goal is not to design a network embedding algorithm that outperforms the state-of-the-art algorithms, it is interesting to examine our model’s ability to predict individual characteristics as a network embedding algorithm. If the learned endowments have a decent predictive power for individual characteristics, we can then believe that we have effectively learned the endowment vectors, which can be used for further analysis such as agent-based modeling. We extract characteristics that are not directly relevant to nodes’ ego network attributes (see Supplementary Note 2 for a full list). We split the nodes and their learned endowment vectors into training (75%) and test (25%) sets. We use support vector machine (SVM) and *k*-nearest neighbors algorithm (*k*-NN) to train the classifiers, and use cross-validation to tune the classifiers’ hyperparameters.

**Fig. 2:**
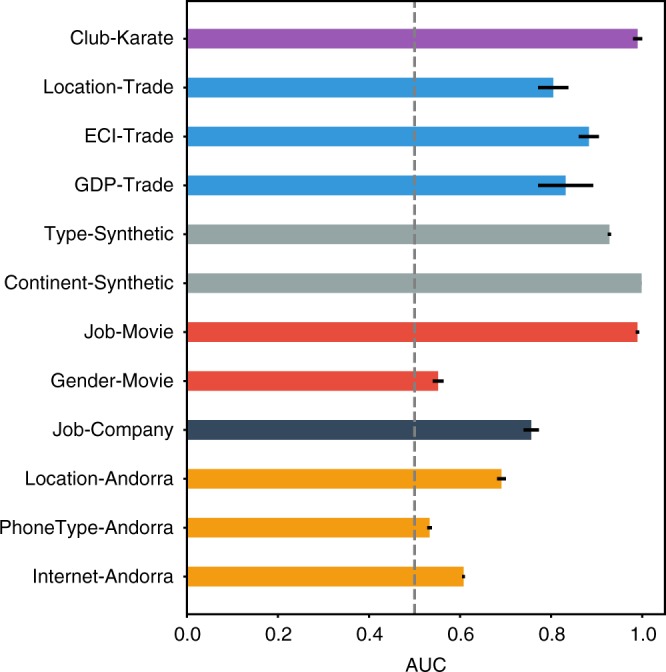
Prediction performance of the learned endowments. We use support vector machine as the classifier. The baseline, random guess algorithm, is indicated by the dashed line. Error bars represent the standard errors for the average AUCs in five random splitting of training-test sets

As shown in Fig. 2, the learned endowment vectors can well predict most individual characteristics by SVM. Note that *k*-NN has similar results in Supplementary Note 5. This result shows that our model can encapsulate the latent features of agents. It is important to highlight that individual characteristics might not be fully reflected in the network; therefore, neither network embedding algorithms nor the present model can guarantee high AUCs for all prediction tasks. However, the learned endowment vectors in fact contain more information than the presented agent features; therefore, they could predict agent characteristics that are not used in this work, e.g., preferences of movie genres.

The accuracy at estimating agent characteristics beyond the input data could be because they are important either in coordination costs (e.g., locations) or exchange benefits (e.g., collaboration between cast members and directors). Some characteristics may have both exchange effects and coordination costs: for example, in a company, subordinates mostly communicate with each other (low coordination costs), but would also interact with their managers occasionally (exchange benefits).

We also compare our results with a network embedding algorithm, DeepWalk^24^, with the same number of dimensions and therefore the same degree of freedom (Supplementary Note 5). Recall that network embedding methods are designed only for dimension reduction; they therefore do not provide economic or sociological insights about the network. Algorithmically, DeepWalk uses an energy function that considers only similarity and not the benefit that can flow from exchanges between agents with very different endowments. Consequently, as might be expected, when our model is compared to DeepWalk, we have better performance if the predicted characteristics are explicitly implied by exchange effects. However, for characteristics explicitly implied by low coordination costs between similar people, the performance of the present model is somewhat lower than that of DeepWalk, probably because DeepWalk considers the similarity between neighbors spanning multiple hops. In sum, the ability to predict agent characteristics shows that our model has learned useful information implicit in the network, and that this implicit information can be used for further agent-based modeling.

### Agent-based modeling

We next analyze the properties of the model as an agent-based model. Because of the high degree of freedom of the present model, any manually input distributions of **W**, **b**, and **c** may appear too arbitrary and do not reflect any real-world situation. We therefore use the learned endowments and parameters as the input to study both micro- and macro- level properties of this model. Our model exhibits many complex and well-known social phenomena, suggesting that these phenomena could be caused by the simple mechanisms of exchange benefits and coordination costs among heterogeneous agents.

At the micro level, an interesting question is how an agent’s endowments will affect their ego networks. In particular, we consider two variables for agents based on our model. The first variable is a quantitative measure of social status that we call “social power”

7 $${\mathrm{social}}\,{\mathrm{power}}(i) = {\mathbf{b}} \cdot {\mathbf{w}}_{\mathbf{i}}.$$

Social power means “the potential for social influence”^49^, or the potential benefits that one could bring to the other. Recall that *b*_*k*_ measures how beneficial the *k*-th dimension is. *w*_*ik*_ is the *i*-th agent’s value on the *k*-th dimension. As *b*_*k*_ × *w*_*ik*_ increases, *i* is more likely to benefit others on the *k*-th dimension. Therefore, it is sensible to represent an agent’s social status by the dot product of **b** and **w**_**i**_. Therefore, the definition of this variable is consistent with the concept, social power. The utility of this social power for social exchange leads naturally to the formation of a network structure, which is often described as hierarchical, especially within the surrounding homophilic group.

The second variable is “social exclusion”, which measures the extent to which an agent is marginalized^50^:

8 $${\mathrm{social}}\,{\mathrm{exclusion}} = \left\| {{\mathbf{c}} \circ {\mathbf{w}}_{\mathbf{i}}} \right\|_2.$$

Recall that we have constrained the means for all dimensions to be 0. If an agent has a large absolute value on some dimension, she is believed to be on the margin of that dimension because a higher cost is needed when she links to another arbitrary person.

**Fig. 3:**
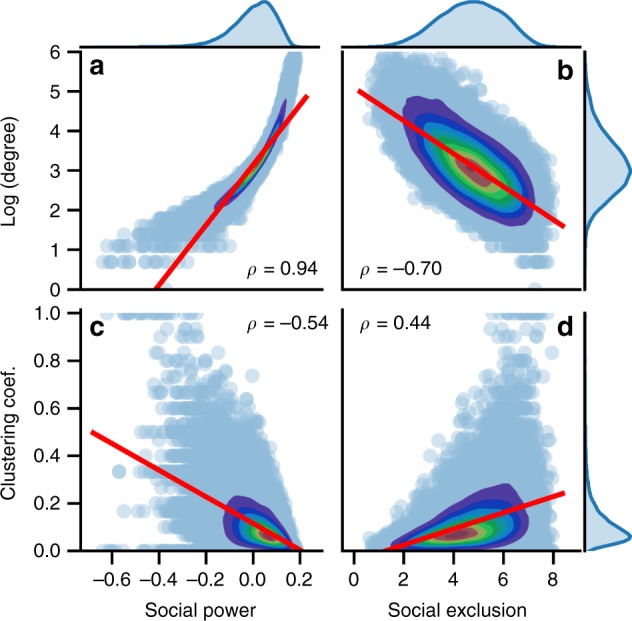
Correlations between endowment-related variables and ego networks statistics for the Andorra dataset. **a** Social power versus degree. **b** Social exclusion versus degree. **c** Social power versus clustering coefficient. **d** Social exclusion versus clustering coefficient. Colors represent the density of data points, where red indicates dense areas and purple indicates sparse areas. All data points are plotted as light blue dots. *ρ* denotes Pearson correlation coefficients. All Pearson correlation coefficients are significant at level *p* < 0.001

We are interested in the correlation between the social power or social exclusion and statistics of their ego networks (i.e., degree and clustering coefficient). The results of the Andorra dataset is presented in Fig. 3, and similar results for other datasets are reported in Supplementary Note 6. We find that “social power” is strongly positively correlated with degree, while “social exclusion” is strongly negatively correlated with degree. This finding is consistent with the implication of the proposed model: people with high (beneficial) endowments can potentially benefit others to a greater degree; people on the margin of the society have fewer opportunities to interact with others. More interestingly, we examine the correlations between social power or exclusion and the clustering coefficients for the nodes. A high clustering coefficient means that the agent’s neighbors are closely connected, and therefore indicates that the agent’s neighbors might lack diversity. We find that people have lower clustering coefficients on the network if they have higher social power or lower social exclusion; that is, high status (power) people have more diverse social networks, a well-known and important aspect of human networks.

The proposed model can also predict macro-level dynamics of networks. As an illustration, we are focused on the impact of the systematic change of cost scaling parameters **c** (i.e., reducing **c** to **c**′ = (1 − *α*)**c**, *α*∈[0, 1]) on the macro statistics of the social network. Decreases in coordination costs are typically caused by advances in information technology (e.g., the Internet) or transportation (e.g., a new railway). We then employ agent-based modeling according to the learned endowment vectors and utility functions to reconstruct the empirical social networks (see Supplementary Note 7 for the approach). Finally, we compute density, average clustering coefficient, average shortest path in the giant component, and interaction diversity (defined as Eq. (9)), where ${\cal E}$ represents the edge set of the network, and **c** is the value after being reduced. Note that here we do not change the relative ratios among *c*_*k*_ (1 ≤ *k* ≤ *K*); it is therefore sensible to incorporate the **c** into Eq. (9) after being normalized by $\left\| {\mathbf{c}} \right\|_2$.

9 $${\mathrm{interaction}}\,{\mathrm{diversity}} = \frac{1}{{|{\cal E}|}}\mathop {\sum}\limits_{(i,j) \in {\cal E}} \frac{{\left\| {{\mathbf{c}} \circ ({\mathbf{w}}_{\mathbf{i}} - {\mathbf{w}}_{\mathbf{j}})} \right\|_2}}{{\left\| {\mathbf{c}} \right\|_2}}$$

**Fig. 4:**
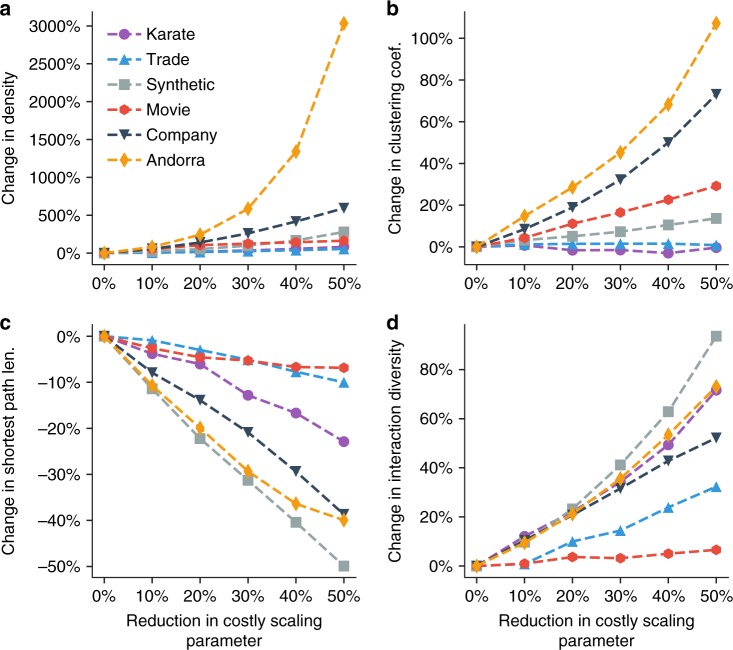
Impact of reduction in costly scaling parameters on macro-level network statistics. **a** Density. **b** Clustering coefficient. **c** Average of shortest path lengths. **d** Interaction diversity. *X*-axis represents the reduction of costly scaling parameters; *y*-axis represents the dynamics of the macro-level network statistics when costly scaling parameters are reduced

Figure 4 shows the impact of reducing **c** on the macro statistics of all networks. We find that as the cost scaling parameters **c** decrease, the density significantly increases while clustering coefficient does not increase much. This indicates that the decrease in coordination costs (e.g., adoption of the Internet) results in more links, and increases social cohesion or balance^51^, i.e., the connectivity between one’s neighbors. The decreasing trend of shortest paths between pairs reveals that the decrease of the coordination cost could diminish the power of social hierarchy. The trend of interaction diversity indicates that the decrease of coordination costs leads to greater connections between more dissimilar individuals. These synthetic findings indicate that the coordination costs’ reduction, usually caused by technology advances, results in a society with less hierarchy and  more opportunities for social connection, especially for dissimilar people.

## Discussion

Inspired by network embedding methods that represent agents by vectors, this study also applies vector representations for heterogeneous agents, referred to as their “endowment vectors”. Our model is more interpretable than network embedding algorithms because we can economically and sociologically explain the link formation mechanism, by the trade-off between the exchange benefits and coordination costs among agents. We learned the endowment vectors from empirical network data, which can be used to predict a variety of other agent properties, and to demonstrate inter-agent network characteristics such as social status and diversity that are well-known from social science literature.

In particular, we highlight the necessity of trading off between beneficial exchange effects and coordination costs. Most link formation models use only one or the other. We show that we can effectively learn the representations for agents from empirical networks by optimization methods that incorporate these trade-offs, without explicitly modeling social status, hierarchy, or the dynamics of social networks. This result suggests that many characteristics that are described in the social science literature are due to the trade-off between coordination costs and exchange benefits, rather than being fundamental effects or biases.

There are several interesting future directions based on this work. First, it is intriguing to consider the influence of existing neighbors on the marginal utilities of adding one more neighbor. For instance, the marginal utility of befriending a person should be higher when an ego has 10 friends than when the ego has 100 friends. Incorporating this interaction effect is difficult because this will require combinatorial optimization methods. Second, it is a promising direction to incorporate an indirect effect: the utility of “friends’ friends”. When we befriend a person, we do not only benefit from this person, but also this person’s friends because we obtain useful information from and have small coordination costs with this person’s friends. The indirect effect is reminiscent of several network embedding methods, including DeepWalk, which embed nodes on randomly sampled paths to have similar representations. Finally, we may take into account broader interaction effects such as “reputation”: when people reach out to an ego, the ego may reciprocate a link even if the link does not directly benefit the ego.

## Methods

### Problem setup

Let ${\cal I} = \{ 1,2,...,N\}$ be a group of *N* and potentially connected agents indexed by *i* (or *j*, *l*). Let *K* be the dimensionality of endowments that drives the formation of the social network of the group, indexed by *k*. Each agent has a latent endowment vector **w**_**i**_ = (*w*_*i*1_,...,*w*_*iK*_)^*T*^, with each dimension indicating an aspect of the individual’s attributes. Let **W** = (**w**_1_,...,**w**_*N*_)^*T*^. We observe all edges among the *N* agents. Let *D* be a set of *N* × *N* adjacency matrices among agents in all periods. *D*_*ij*_ is binary ({0,1}). *D*_*ij*_ = 1 if there is an edge from *i* to *j*, and *D*_*ij*_ = 0 otherwise. For the convenience of showing pairwise stability, the study is restricted to undirected graphs, i.e., *D*_*ij*_ = *D*_*ji*_.

Agents make rational choices by comparing their endowment vectors with potential friends. Agents maximize their utility functions ($U_i:2^{{\cal I}/\{ i\} } \to {\Bbb R}$ for each *i*) dependent on the differences between their endowment vectors and all possible candidates (all other agents). *U*_*i*_ is also parameterized by **W**, **b**, and **c**. Δ*u*_*i*_(*j*) is the marginal utility that *j* brings to *i*. We therefore predict *D*_*ij*_ by Δ*u*_*i*_(*j*).

### Data description

- Andorra. We collected the nationwide call detail records in Andorra from July 2015 to June 2016. Utilizing the country code, we filtered out all non-citizens, leaving 32,829 citizens with at least one call interactions with another. If the (*i*,*j*) had at least one effective call (duration greater than 0 s), we set *D*_*ij*_ = *D*_*ji*_ = 1; otherwise *D*_*ij*_ = *D*_*ji*_ = 0. This process results in 513,931 links. To demonstrate the effectiveness of the learned endowments, we also extracted three characteristics of individuals: phone type, frequent city, and Internet usage. The phone type was identified by the type allocation code, and we classified each type into Apple, Samsung, and others (the distribution of three types is balanced). For each phone number, we employed the last phone type that we observed. Note that type phone is strongly correlated with important individual characteristics such as income. The most frequent city was identified by the cell tower id. We classified each phone number by the location where it shows up most frequently throughout the year, this location is thus likely the work location of the individual (some individuals’ work location may be their home). Internet usage was computed by the total duration of cellular data. In the prediction task, we classified Internet usage into high (more than median) and low (less or equal than median). Details of the datasets, such as statistics of individual characteristics and network degree distribution, are shown in Description in detail in Supplementary Note 2.
- Movie. To highlight the exchange effects, we examine a specific type of social network, director-cast movie collaboration network, where a node represents either a movie director or an actor/actress, and an edge between a director *i* and an actor/actress *j* represents a collaboration between *i* and *j*. *D*_*ij*_ = *D*_*ji*_ = 1 means that *i* and *j* collaborated at least once; 0 otherwise. Note that the social network is close to a bipartite graph where nodes are partitioned into directors and cast (some people have both cast and director experience). We extracted 3493 movies throughout 2000–2016, and retained individuals with at least five movies within this period, resulting in 160 directors and 2628 cast members, and 10,399 director-cast pairs. To validate the effectiveness of the learned endowments, we extracted two individual characteristics: occupation and gender. For occupation, we labeled an individual as a director if she functioned as a director in more than a half of the movies in which she engaged; cast otherwise. For gender, we collected 1840 males and 761 females and 186 unlabeled.
- Synthetic. We manually establish a network of 2500 agents. Agents are indexed by (*x*,*y*) (*i* = 50*x* + *y*), 0 ≤ *x* ≤ 49, 0 ≤ *y* ≤ 49, $x,y \in {\Bbb N}$. Each agent therefore resides at a unique location on the 50 × 50 grid, and the agent has a probability of 0.5 to be either type A (e.g., a buyer) or type B (e.g., a seller). Buyers (sellers) are exploring sellers (buyers) in their neighborhood with Manhattan distance ≤3. The network is therefore a bipartite graph where buyers and sellers exchange goods and money. This data generating process results in 14,453 edges. We predict the type and location (divide the plane into four parts) for all agents.
- Company. A network of employees in a company where edges represent a call and text communication (MobileD in^52^). Each employee is labeled as a manager or a subordinate. In total, we have 420 managers and 1564 subordinates, with 12,751 edges among them. In this network, managers are mostly connected with managers and subordinates are mostly connected with subordinates. At the same time, subordinates also interact with their respective managers occasionally. We believe that this dataset should show a trade-off between coordination and exchange; for example, managers and subordinates have exchange effects, and they have lower coordination costs to interact with the same type.
- Trade. We use the 2014 international trade data provided by the United Nations Statistical Division (UN Comtrade Database: [https://comtrade.un.org/]), specifically the cleaned version provided by the BACI team using their own methodology of harmonization^53^. We created a network of countries, where an edge indicates that the trade value between two countries is >1 billion dollars (for both directions). This process resulted in 100 countries with at least one link, and 703 undirected edges among them. We predict the GDP, economic complexity index (ECI)^54^, and the countries’ continents for this dataset.

### Details in learning

For computational simplicity and better fitting performance (see Supplementary Note 8), we split the dimensions into “beneficial dimensions” and “costly dimensions”. In Eq. (5), every dimension (say the *k*-th) can contribute to both benefits and costs if both *b*_*k*_ and *c*_*k*_ are greater than zero. However, it is not difficult to see that if we constrain some dimensions to have zero-valued beneficial scaling parameters (*b*_*k*_ = 0) or costly scaling parameters (*c*_*k*_ = 0), the dimensionality of the model (*K*) will increase but the capacity of data fitting will not change. During the learning process, a connected pair (*i*, *j*) may result in either an increase in the difference on some beneficial dimension (with *b*_*k*_ > 0) or a decrease in the difference on some costly dimension (with *c*_*k*_ > 0) between their endowment vectors. Empirically, if both *b*_*k*_ and *c*_*k*_ are positive, these two conflicting effects (to increase or to decrease the utility on the same dimension) would hinder an effective convergence (shown in Supplementary Note 8); we conjecture that this is because we are optimizing a non-linear non-convex loss function. Therefore, we separate the *K* dimension into *K*_bnf_ “beneficial dimensions” and *K*_cst_ “costly dimensions” (*K*_bnf_ + *K*_cst_ = *K*). By comparing the performances of link fitting for different *K*_bnf_ and *K*_cst_, we select the optimal $K_{{\mathrm{bnf}}}^ \ast$ and $K_{{\mathrm{cst}}}^ \ast$, and consequently K^*^. For simplicity, we let *b*_*k*_ = 0, for *k* > *K*_bnf_; and *c*_*k*_ = 0, for *k* ≤ *K*_bnf_. ${\boldsymbol{\theta }} = \left( {b_1,b_2,...,b_{K_{{\mathrm{bnf}}}},c_{K_{{\mathrm{bnf}}} + 1},c_{K_{{\mathrm{bnf}}} + 2},...,c_K} \right)$. In Supplementary Note 8, we show empirically that the performances of link fitting and node classifications are worse when we do not split dimensions into beneficial and costly dimensions; and that even when we do not split dimensions, the learning algorithm will lead most dimensions to be either “beneficial” or “costly”, i.e., either *b*_*k*_ or *c*_*k*_ is very close to zero. More details can be found in Supplementary Note 3.

### Code Availability

Code is available online: https://github.com/yuany94/endowment.

## Electronic supplementary material

Supplementary Information

## Data Availability

The network data and individual attributes are available online: https://github.com/yuany94/endowment.

## References

[1] Newman, M. *Networks: an introduction* (Oxford Univ. Press, Oxford, 2010).

[2] Wasserman, S. & Faust, K. *Social network analysis: methods and applications*, vol. 8 (Cambridge Univ. Press, Cambridge, 1994).

[3] Jackson MO (2005). A survey of network formation models: stability and efficiency. Group Formation in Economics: Networks, Clubs, and Coalitions.

[4] Borgatti SP, Mehra A, Brass DJ, Labianca G (2009). Network analysis in the social sciences. Science.

[5] Rogers, E. M. *Diffusion of innovations* (Simon and Schuster, New York City, 2010).

[6] Bakshy, E., Rosenn, I., Marlow, C. & Adamic, L. The role of social networks in information diffusion. In *Proc. 21st International Conference on World Wide Web*, 519–528 (ACM, Lyon, France, 2012).

[7] Fowler JH, Christakis NA (2008). Dynamic spread of happiness in a large social network: longitudinal analysis over 20 years in the framingham heart study. BMJ.

[8] Banerjee A, Chandrasekhar AG, Duflo E, Jackson MO (2013). The diffusion of microfinance. Science.

[9] Fiorina MP, Abrams SJ (2008). Political polarization in the american public. Annu Rev. Polit. Sci..

[10] Aral S, Muchnik L, Sundararajan A (2009). Distinguishing influence-based contagion from homophily-driven diffusion in dynamic networks. Proc. Natl Acad. Sci. USA.

[11] Meyers LA, Newman M, Pourbohloul B (2006). Predicting epidemics on directed contact networks. J. Theor. Biol..

[12] Christakis NA, Fowler JH (2007). The spread of obesity in a large social network over 32 years. N. Engl. J. Med..

[13] Bond RM (2012). A 61-million-person experiment in social influence and political mobilization. Nature.

[14] Watts DJ, Strogatz SH (1998). Collective dynamics of ‘small-world’ networks. Nature.

[15] Barabási AL (2009). Scale-free networks: a decade and beyond. Science.

[16] Jackson MO, Wolinsky A (1996). A strategic model of social and economic networks. J. Econ. Theory.

[17] Skyrms B., Pemantle R. (2000). A dynamic model of social network formation. Proceedings of the National Academy of Sciences.

[18] Ohtsuki H, Hauert C, Lieberman E, Nowak MA (2006). A simple rule for the evolution of cooperation on graphs and social networks. Nature.

[19] Nowak MA (2006). Five rules for the evolution of cooperation. Science.

[20] Jackson, M. O. *Social and economic networks* (Princeton Univ. Press, Princeton, 2010).

[21] Mele A (2017). A structural model of dense network formation. Econometrica.

[22] Christakis, N. A., Fowler, J. H., Imbens, G. W. & Kalyanaraman, K. An empirical model for strategic network formation, Preprint at http://www.nber.org/papers/w16039 (2010).

[23] Chandrasekhar, A. G. & Jackson, M. O. A network formation model based on subgraphs, Preprint at https://arxiv.org/abs/1611.07658 (2016).

[24] Perozzi, B., Al-Rfou, R. & Skiena, S. Deepwalk: Online learning of social representations. In *Proc. 20th ACM SIGKDD International Conference on Knowledge Discovery and Data Mining*, 701–710 (ACM, New York City, USA, 2014).

[25] Tang, J. et al. Line: large-scale information network embedding. In *Proc. 24th International Conference on World Wide Web*, 1067–1077 (ACM, Florence, Italy, 2015).

[26] Grover, A. & Leskovec, J. Node2vec: scalable feature learning for networks. In *Proc. 22nd ACM SIGKDD International Conference on Knowledge Discovery and Data Mining*, 855–864 (ACM, San Francisco, USA, 2016). 10.1145/2939672.2939754 PMC5108654 27853626

[27] Kipf, T. N. & Welling, M. Semi-supervised classification with graph convolutional networks. In *Proc. 5th International Conference on Learning Representations* (ACM, Toulon, France, 2017).

[28] McKane, A. J. & Drossel, B. Models of food web evolution. *Ecological Networks: linking Structure to Dynamics in Food Webs* 223–243 (Oxford Univ. Press, Oxford, 2006).

[29] Airoldi EM, Blei DM, Fienberg SE, Xing EP (2008). Mixed membership stochastic blockmodels. J. Mach. Learn. Res..

[30] Jackson MO, Xing Y (2014). Culture-dependent strategies in coordination games. Proc. Natl Acad. Sci. USA.

[31] McPherson M, Smith-Lovin L, Cook JM (2001). Birds of a feather: homophily in social networks. Annu. Rev. Sociol..

[32] Mas-Colell A (1995). Microeconomic theory.

[33] Rogers EM, Bhowmik DK (1970). Homophily-heterophily: relational concepts for communication research. Public Opin. Q..

[34] Johnson NF (2009). Human group formation in online guilds and offline gangs driven by a common team dynamic. Phys. Rev. E.

[35] Kimura D, Hayakawa Y (2008). Coevolutionary networks with homophily and heterophily. Phys. Rev. E.

[36] Alpert MI, Anderson WT (1973). Optimal heterophily and communication effectiveness: some empirical findings. J. Commun..

[37] Boguná M, Pastor-Satorras R, Daz-Guilera A, Arenas A (2004). Models of social networks based on social distance attachment. Phys. Rev. E.

[38] Currarini S, Jackson MO, Pin P (2009). An economic model of friendship: homophily, minorities, and segregation. Econometrica.

[39] Cook KS, Yamagishi T (1992). Power in exchange networks: a power-dependence formulation. Soc. Network.

[40] Friedkin NE (1992). An expected value model of social power: predictions for selected exchange networks. Soc. Network..

[41] Kleinberg, J. & Tardos, É. Balanced outcomes in social exchange networks. In *Proc. 40th Annual ACM Symposium on Theory of Computing*, 295–304 (ACM, Victoria, Canada, 2008).

[42] Watson WE, Kumar K, Michaelsen LK (1993). Cultural diversity’s impact on interaction process and performance: comparing homogeneous and diverse task groups. Acad. Manag. J..

[43] Hong L, Page SE (2004). Groups of diverse problem solvers can outperform groups of high-ability problem solvers. Proc. Natl Acad. Sci. USA.

[44] Page, S. E. *The difference: how the power of diversity creates better groups, firms, schools, and societies* (Princeton Univ. Press, Princeton, 2008).

[45] Abadi, M. & TensorFlow, A. A. B. P. Large-scale machine learning on heterogeneous distributed systems. In *Proc. 12th USENIX Symposium on Operating Systems Design and Implementation*, 265-283 (USENIX, Savannah, USA, 2016).

[46] Do AL, Rudolf L, Gross T (2010). Patterns of cooperation: fairness and coordination in networks of interacting agents. New J. Phys..

[47] Kingma, D. & Ba, J. Adam: a method for stochastic optimization. In *Proc. 3rd International Conference on Learning Representations* (ACM, San Diego, USA, 2015).

[48] Zachary WW (1977). An information flow model for conflict and fission in small groups. J. Anthropol. Res..

[49] French JR, Raven B, Cartwright D (1959). The bases of social power. Class. Organ. Theory.

[50] Strauss RS, Pollack HA (2003). Social marginalization of overweight children. Arch. Pediatr. Adolesc. Med..

[51] Cartwright D, Harary F (1956). Structural balance: a generalization of heider’s theory. Psychol. Rev..

[52] Tang, J., Lou, T., Kleinberg, J. & Wu, S. Transfer link prediction across heterogeneous social networks. *ACM Trans Inf Syst* **9,**Article 43 (2010).

[53] Gaulier, G. & Zignago, S. Baci: international trade database at the product-level (the 1994–2007 version) https://ideas.repec.org/p/cii/cepidt/2010-23.html (2010).

[54] Hidalgo CA, Hausmann R (2009). The building blocks of economic complexity. Proc. Natl Acad. Sci. USA.

