# Supplementary material for: An interpretable approach for social network formation among heterogeneous agents
Source: Nat Commun. 2018 Nov 8;9:4704. doi: 10.1038/s41467-018-07089-x (PMC6224571; doi:10.1038/s41467-018-07089-x)
Supplement: Supplementary file 1 — Supplementary Information [file 41467_2018_7089_MOESM1_ESM.pdf]

Supplementary Information

An Interpretable Approach for Social Network Formation Among Heterogeneous Agents

Yuan et al.

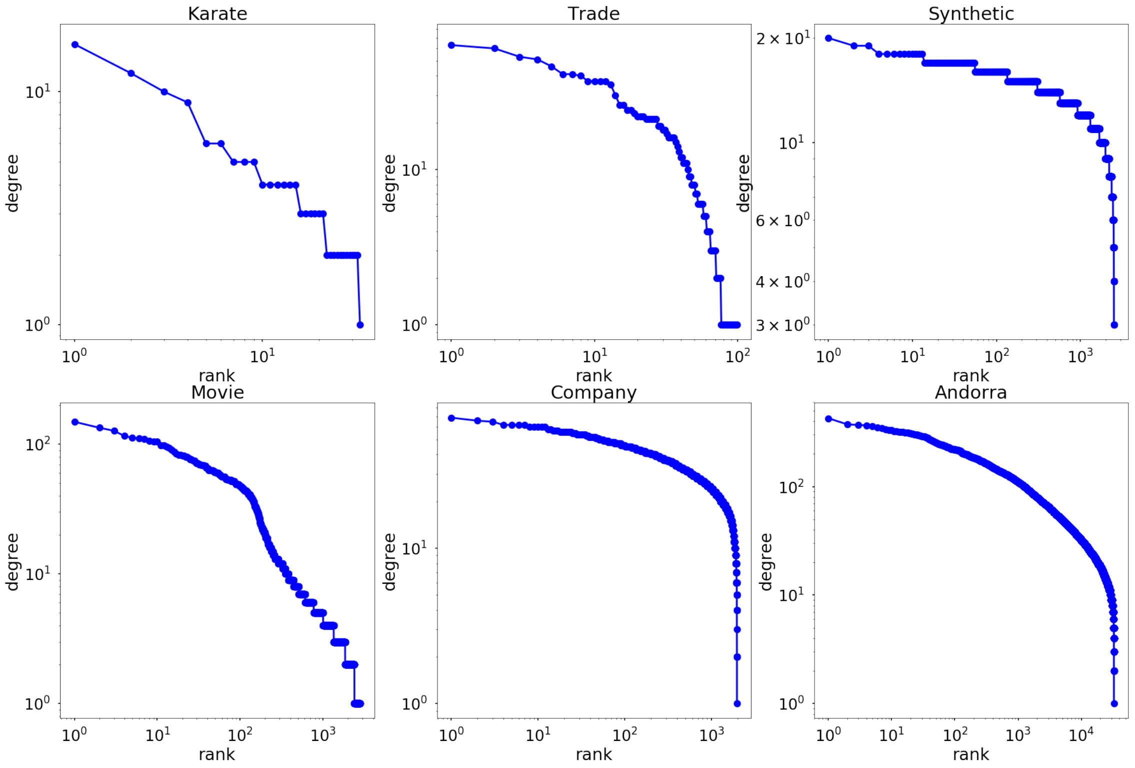

Supplementary Figure 1. Degree distribution for all datasets.

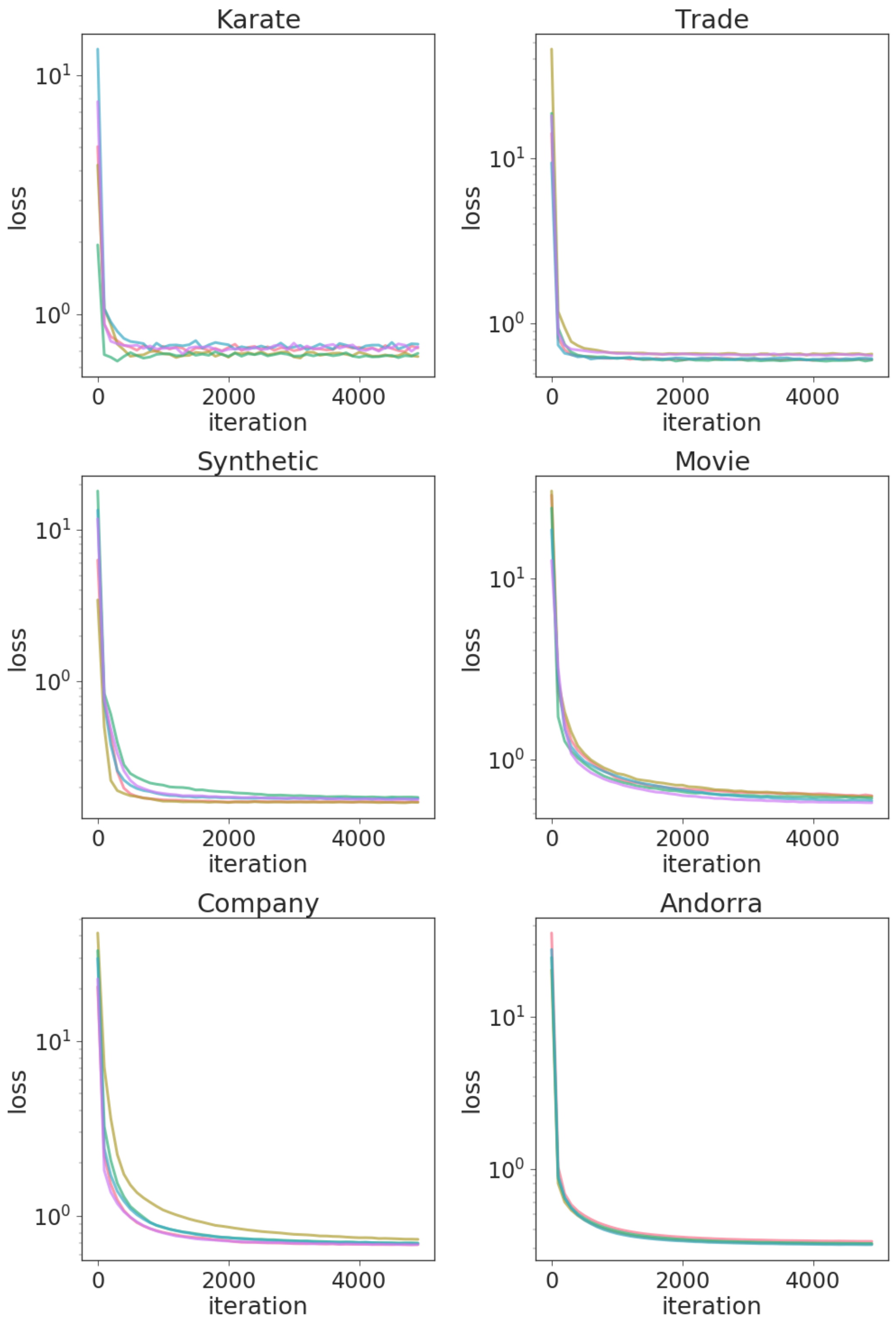

Supplementary Figure 2. Learning curves for losses.

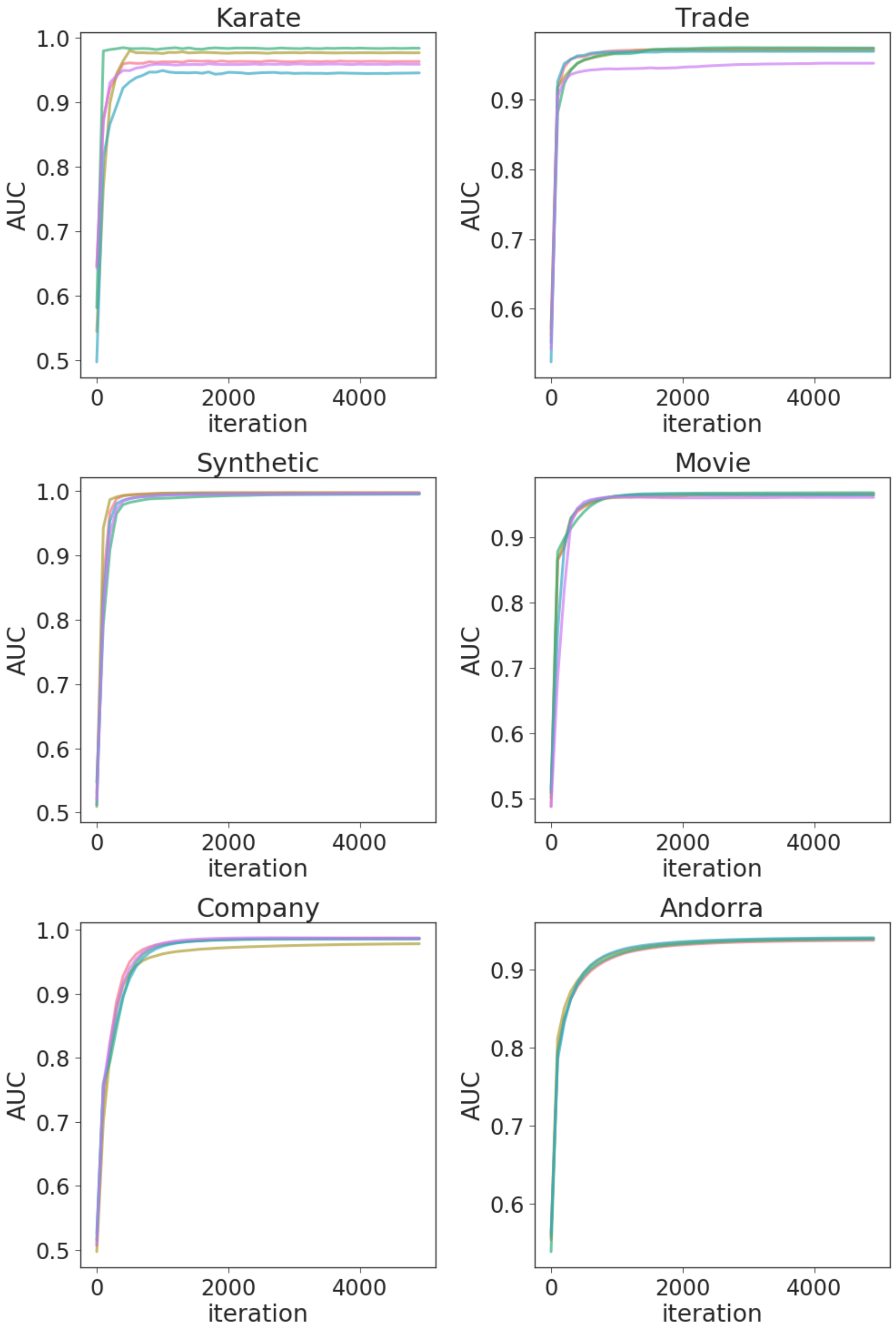

Supplementary Figure 3. Learning curves for AUCs.

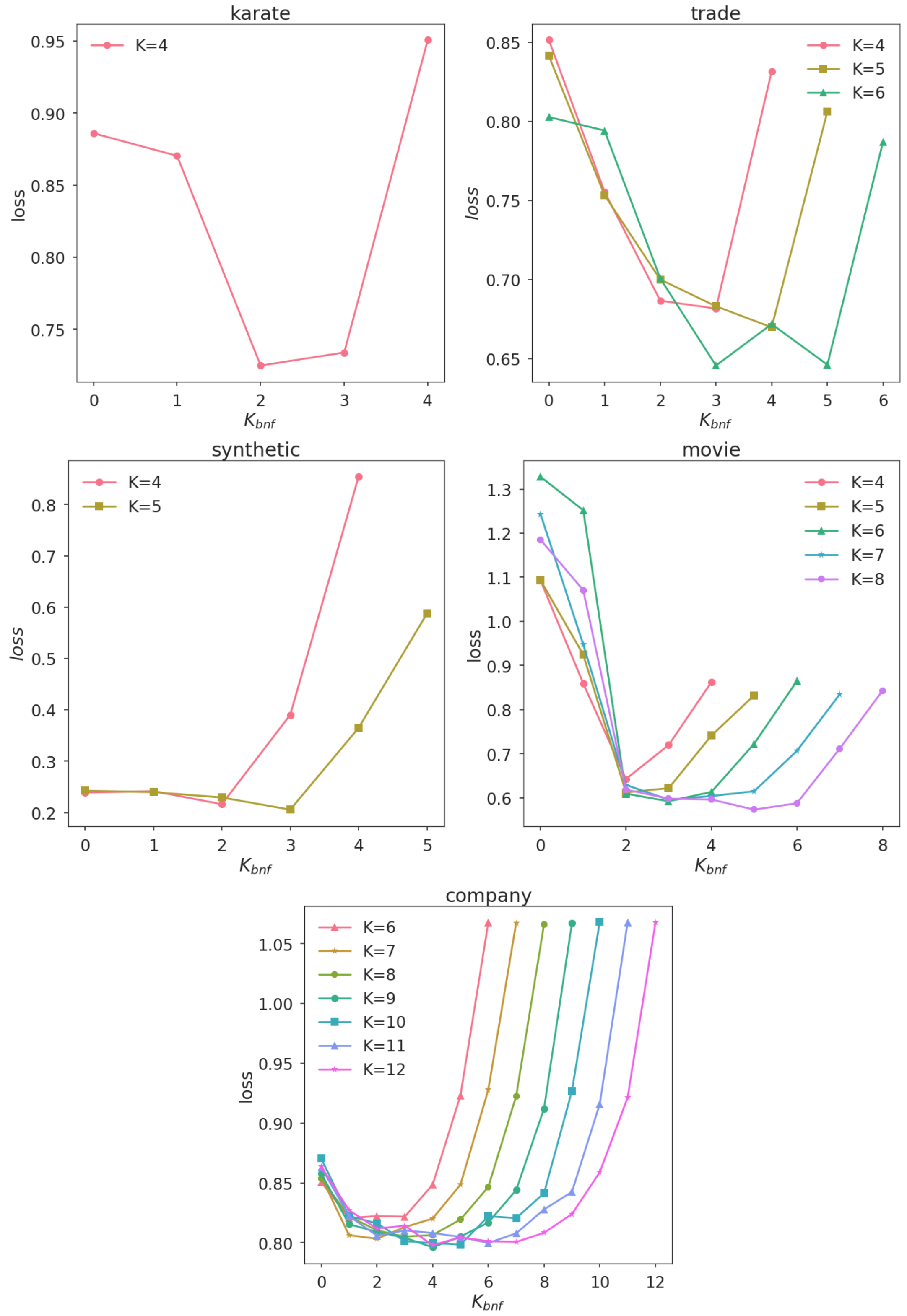

Supplementary Figure 4. Learning losses for K and K_{bnf}.

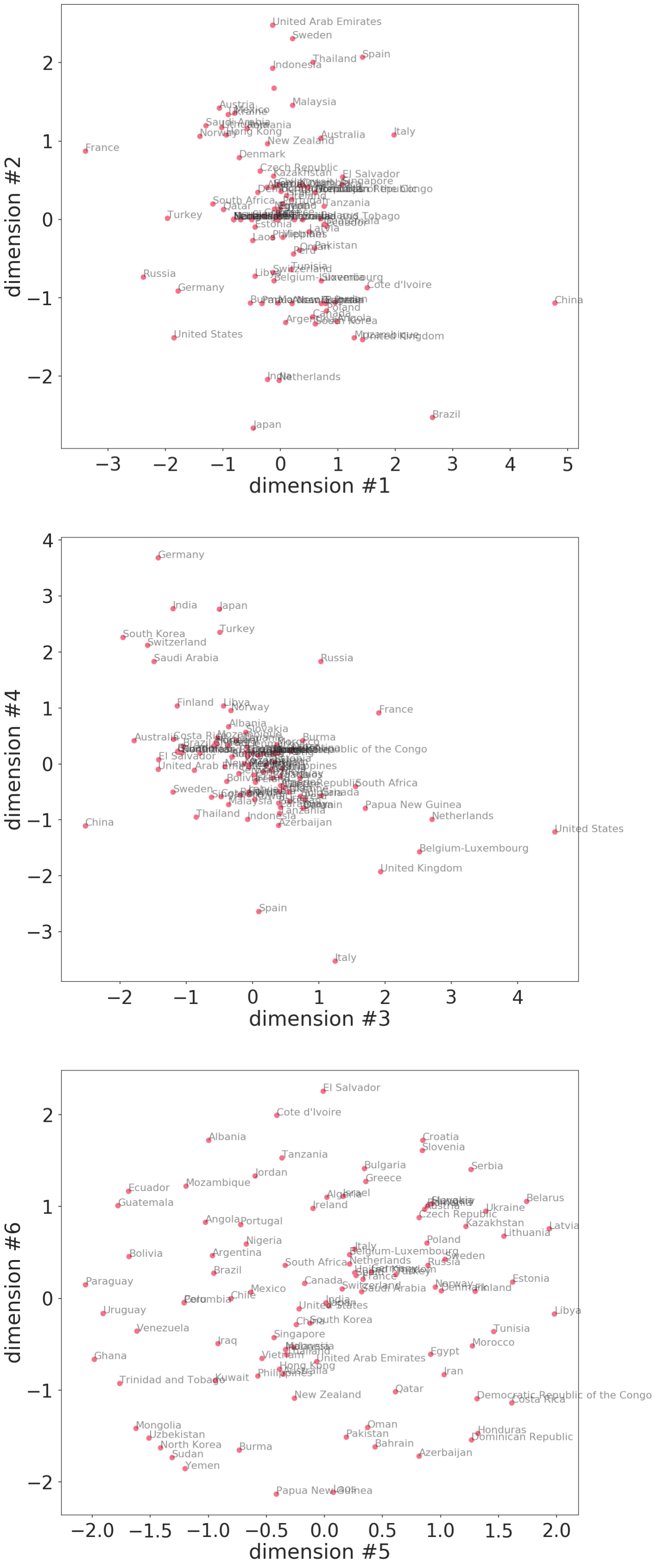

Supplementary Figure 5. Illustration of learned endowments for trade network. We let $(K, K_{\text{bnf}}) = (6, 4)$ for visualization. The first four dimensions are beneficial dimensions and the last two dimensions are costly dimensions.

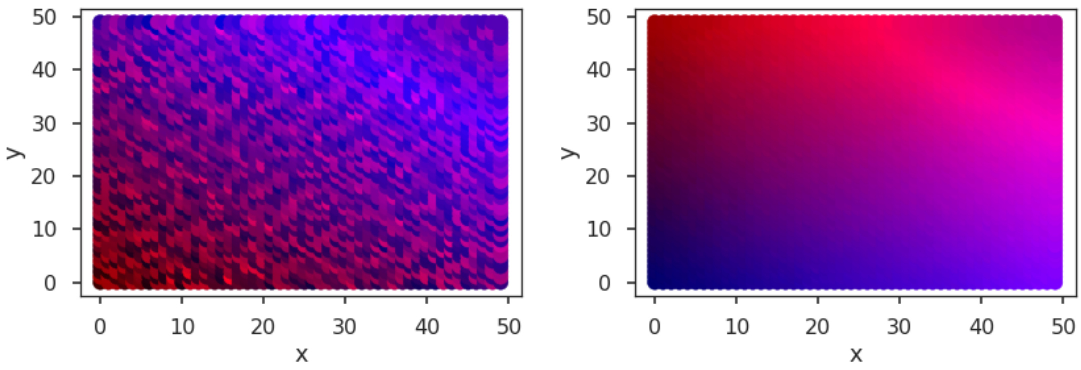

Supplementary Figure 6. Learned endowment vectors for agent i, $i = 50x + y$. The left panel shows the two beneficial endowments, while the right panel shows the two costly endowments. Colors indicate the value on respective dimensions: red for the first and third dimensions and blue for the second and fourth dimensions. Purple indicates high value on both dimensions; black indicates low value on both dimensions.

Supplementary Table 1. Statistics of Individual Characteristics in Andorra CDR

Phone Type	
Samsung	14,609
Apple	9,422
Others	8,798
Location Cluster	
Centre	20,830
St Julia	3,377
Massana	3,703
Arans	264
Encamp	3,641
Soldeu	536
Pas De La Cas	478
Internet usage (hour)	
0%	0.00
25%	5.95
50%	1,076.28
75%	3,088.07
100%	8,746.29

Supplementary Table 2. Prediction performance of the present model and DeepWalk. Standard errors of the means in five classifications are reported in parentheses.

Dataset	Present-SVM	Present-KNN	DeepWalk-SVM	DeepWalk-KNN
Club-Karate	0.9900	0.9894	1.0000	1.0000
	(0.0100)	(0.0065)	(0.0000)	(0.0000)
Location-Trade	0.8045	0.8125	0.8597	0.9099
	(0.0333)	(0.0337)	(0.0439)	(0.0215)
ECI-Trade	0.8828	0.9098	0.8750	0.8504
	(0.0221)	(0.0186)	(0.0272)	(0.0257)
GDP-Trade	0.8319	0.7633	0.7355	0.7459
	(0.0607)	(0.0697)	(0.0622)	(0.0680)
Type-Synthetic	0.9279	0.9446	0.5340	0.4470
	(0.0036)	(0.0048)	(0.0081)	(0.0106)
Location-Synthetic	0.9986	0.9908	0.9971	0.9987
	(0.0001)	(0.0016)	(0.0021)	(0.0008)
Job-Company	0.8538	0.8549	0.9491	0.9352
	(0.0098)	(0.0075)	(0.0061)	(0.0040)
Job-Movie	0.9931	0.9854	0.5774	0.4824
	(0.0008)	(0.0045)	(0.0187)	(0.0041)
Gender-Movie	0.5445	0.5266	0.5546	0.5294
	(0.0105)	(0.0006)	(0.0140)	(0.0075)
Location-Andorra	0.6908	0.7141	0.8265	0.8215
	(0.0097)	(0.0064)	(0.0058)	(0.0084)
PhoneType-Andorra	0.5331	0.5592	0.5918	0.5805
	(0.0052)	(0.0019)	(0.0034)	(0.0049)
Internet-Andorra	0.6075	0.5822	0.6493	0.6446
	(0.0030)	(0.0305)	(0.0022)	(0.0070)

Supplementary Table 3. Pearson correlation coefficients between social power or exclusion and degree or clustering coefficients. *: $p < 0.1$; **: $p < 0.01$.

Dataset	Karate	Trade	Synthetic	Movie	Company
social power vs log(degree)	0.713**	-0.179*	0.406**	0.808**	-0.011
social exclusion vs log(degree)	-0.365*	-0.833**	-0.257**	-0.593 **	-0.675**
social power vs clustering coef.	-0.583**	-0.345**	-	-	0.007
social exclusion vs clustering coef.	0.368*	-0.636**	-	-	-0.007

Supplementary Table 4. The optimal AUC in five runs in the “split” and “non-split” conditions.

**Condition**	**Karate**	**Trade**	**Synthetic**	**Movie**	**Company**	**Andorra**
Split	0.9848	0.9685	0.9992	0.9608	0.9870	0.9476
Non-Split	0.9245	0.9537	0.9957	0.9611	0.9321	0.9318

Supplementary Table 5. Comparison of predictive power of learned endowments on "split" and "non-split" conditions.

Dataset	Split-SVM	Split-KNN	Nonsplit-SVM	Nonsplit-KNN
Club-Karate	0.9900	0.9894	0.9089	0.9189
	(0.0100)	(0.0065)	(0.0365)	(0.0308)
Location-Trade	0.8045	0.8125	0.8140	0.8849
	(0.0333)	(0.0337)	(0.0222)	(0.0421)
ECI-Trade	0.8828	0.9098	0.9050	0.9093
	(0.0221)	(0.0186)	(0.0267)	(0.0322)
GDP-Trade	0.8319	0.7633	0.7833	0.7843
	(0.0607)	(0.0697)	(0.0672)	(0.0653)
Type-Synthetic	0.9279	0.9446	0.9346	0.9541
	(0.0036)	(0.0048)	(0.0044)	(0.0017)
Location-Synthetic	0.9986	0.9908	0.9986	0.9890
	(0.0001)	(0.0016)	(0.0001)	(0.0008)
Job-Company	0.8538	0.8549	0.6745	0.6442
	(0.0098)	(0.0075)	(0.0077)	(0.0123)
Job-Movie	0.9931	0.9854	0.9926	0.9864
	(0.0008)	(0.0045)	(0.0010)	(0.0025)
Gender-Movie	0.5445	0.5266	0.5199	0.5770
	(0.0105)	(0.0006)	(0.0109)	(0.0021)
Location-Andorra	0.6908	0.7141	0.5315	0.6215
	(0.0097)	(0.0064)	(0.0336)	(0.0116)
PhoneType-Andorra	0.5331	0.5592	0.4880	0.5463
	(0.0052)	(0.0019)	(0.0100)	(0.0067)
Internet-Andorra	0.6075	0.5822	0.5107	0.5889
	(0.0030)	(0.0305)	(0.0316)	(0.0104)

Supplementary Table 6. Number of beneficial and costly dimensions in the non-split condition.

Dataset	$b_k, c_k = 0$	$b_k = 0,\ c_k > 0$	$b_k = 0,\ c_k < 0$	$b_k, c_k > 0$
Karate	0	1	2	0
Trade	0	2	3	0
Synthetic	0	2	2	0
Company	1	5	5	1
Movie	0	4	2	2
Andorra	0	5	11	0

Supplementary Table 7. The optimal AUC in five runs for variations of the functional form.

**Condition**	**Karate**	**Trade**	**Synthetic**	**Movie**	**Company**	**Andorra**
Present model	0.9848	0.9685	0.9992	0.9608	0.9870	0.9476
Smooth	0.9727	0.9675	0.9885	0.9567	0.9943	0.9395
Abs (ℓ_1)	0.9730	0.9722	0.9907	0.9717	0.9923	0.9379

Supplementary Table 8. Comparison of the prediction performances between the present model and variations. Standard errors are reported in parentheses.

Dataset	Present-SVM	Present-KNN	Abs-SVM	Abs-KNN	Smooth-SVM	Smooth-KNN
Club-Karate	0.9900	0.9894	1.0000	0.9900	0.9900	0.9600
	(0.0100)	(0.0065)	(0.0000)	(0.0100)	(0.0100)	(0.0127)
Location-Trade	0.8045	0.8125	0.6939	0.7656	0.7594	0.7636
	(0.0333)	(0.0337)	(0.0313)	(0.0474)	(0.0215)	(0.0236)
ECI-Trade	0.8828	0.9098	0.7789	0.7562	0.8834	0.8399
	(0.0221)	(0.0186)	(0.0306)	(0.0533)	(0.0296)	(0.0362)
GDP-Trade	0.8319	0.7633	0.8768	0.8601	0.8730	0.8633
	(0.0607)	(0.0697)	(0.0512)	(0.0621)	(0.0568)	(0.0461)
Type-Synthetic	0.9279	0.9446	0.8970	0.9200	0.9307	0.9147
	(0.0036)	(0.0048)	(0.0052)	(0.0041)	(0.0037)	(0.0060)
Location-Synthetic	0.9986	0.9908	0.9985	0.9905	0.9514	0.9415
	(0.0001)	(0.0016)	(0.0002)	(0.0012)	(0.0014)	(0.0044)
Job-Company	0.8538	0.8549	0.77589	0.7657	0.8044	0.7814
	(0.0098)	(0.0075)	(0.0158)	(0.0082)	(0.0102)	(0.0071)
Job-Movie	0.9931	0.9854	0.9882	0.9795	0.9861	0.9774
	(0.0008)	(0.0045)	(0.0011)	(0.0048)	(0.0043)	(0.0042)
Gender-Movie	0.5445	0.5266	0.5011	0.5297	0.5043	0.5526
	(0.0105)	(0.0006)	(0.0086)	(0.0057)	(0.0096)	(0.0094)
Location-Andorra	0.6908	0.7141	0.5840	0.6033	0.6205	0.6395
	(0.0097)	(0.0064)	(0.0191)	(0.0089)	(0.0049)	(0.0054)
PhoneType-Andorra	0.5331	0.5592	0.5608	0.5631	0.5456	0.5654
	(0.0052)	(0.0019)	(0.0053)	(0.0061)	(0.0014)	(0.0030)
Internet-Andorra	0.6075	0.5822	0.6096	0.6122	0.5993	0.6176
	(0.0030)	(0.0305)	(0.0073)	(0.0103)	(0.0112)	(0.0053)

Supplementary Note 1: Pairwise Stability

The proof of the Proposition (1) is derived directly from the definition of pairwise stability. On the one hand, removing an agent in S_i^* leads to a decrease in U_i, i.e.,

$$U_i(S_i^*; \mathbf{W}, \mathbf{b}, \mathbf{c}) \geq U_i(S_i^*/\{j\}; \mathbf{W}, \mathbf{b}, \mathbf{c}), \text{ for } j \in S_i^*. \tag{1}$$

In our specific form (equation (5) in main text), we have

$$\Delta u_i(j) \geq 0, \text{ for } j \in S_i^*. \tag{2}$$

On the other hand, forming a new link does not increase the utility of both agents at the same time, i.e., if

$$U_i(S_i^*; \mathbf{W}, \mathbf{b}, \mathbf{c}) \leq U_i(S_i^* \cup \{j\}; \mathbf{W}, \mathbf{b}, \mathbf{c}), \text{ for } j \notin S_i^*, \tag{3}$$

then

$$U_j(S_j^*; \mathbf{W}, \mathbf{b}, \mathbf{c}) > U_j(S_j^* \cup \{i\}; \mathbf{W}, \mathbf{b}, \mathbf{c}), \text{ for } i \notin S_j^*. \tag{4}$$

Equivalently, between $\Delta u_i(j)$ and $\Delta u_j(i)$, at least one should be less than zero. Therefore, we have

$$\min\big(\Delta u_i(j), \Delta u_j(i)\big) < 0, \text{ for } i \notin S_j^* \text{ and } j \notin S_i^*. \tag{5}$$

Supplementary Note 2: Data description

Here we describe in detail the datasets that we utilize, with a focus on the individual characteristics to predict. Degree distributions are presented in Supplementary Figure 1.

Description of Andorra Dataset

We begin with a brief illustration of the format of the dataset. For each entry, we have the anonymized phone number which initiates a record (call/text/cellular), the starting time of the record, the duration of the record, the cell tower id of the

initiator, the type of the record (call/text/cellular), cell phone carrier (containing country code), the anonymized phone number that receives the call or text, the type allocation code (containing phone type). The statistics of individual characteristics are presented in Supplementary Table 1. Because we do not have fine-grained information for users, we use the following three variables as the proxy variables for individual characteristics.

- **Phone type**. In Andorra, the first and second largest phone types are Samsung and Apple, respectively. Therefore, we distinguish these two phone types from other phone types. We believe that phone type should be (weakly) correlated to individual income and social status; therefore we employ the phone type as one of the variables to predict.
- **Location**. Andorra Telecom records the cell tower location to which each call connects. We are then able to label each user by the place where she appears most frequently. Andorra Telecom classifies cell towers into seven clusters (as shown in Supplementary Table 1). Note that the clusters do not strictly correspond to the seven districts in Andorra.
- **Internet usage**. For Internet usage, we show the minimum, the first quartile, median, the third quartile, and the maximum in the dataset. Youths tend to use the Internet more frequently than old people. Thus Internet usage should be a decent proxy for age since we lack sufficient fine-grained characteristics for the individuals.

Description of Movie Dataset

The TMDB is a public dataset[1]. In the dataset, we have 4,803 movies in total. For each movie, participants are classified into either cast or crew. Each individual is identified by a unique ID. We only extract the actors/actresses and directors, and then establish links between the director and the cast, meaning that "the director invited the actor/actress to the collaboration and the actor/actress agreed". If an individual serves as the director in more than half of the movies that she has engaged

[1] https://www.kaggle.com/tmdb/tmdb-movie-metadata

in, then we label her as “director”, otherwise “actor/actress”. The gender of each individual is also identified in the dataset.

Description of Company Dataset

The company dataset is also a public dataset (MobileD [2]). We examine this network because it shows the collaboration within a company and provides the labels of individual characteristics (managers or subordinates). In this network, managers' neighbors are mostly managers and subordinates' neighbors are mostly subordinates. Therefore, strong homophily (caused by reduced coordination costs) exists on the “job” dimension in this network but there are also exchanges between these two types of employees. We predict whether an employee is a manager or a subordinate.

Description of Trade Dataset

Because we do not consider the strength of a link, we will have a very densely connected network if we do not filter out some links. We filtered out links with export amounts lower than one billion; countries with high trade volumes would retain more links. We also remove countries without any link because then we have no information to estimate information for these countries. This process yields a network of 100 countries.

We extract three features for the 100 countries (or region) in the network:

1. Continent. We predict whether a country belongs to Africa, America, Asia/Pacific and Europe, and measure the performance by the average AUC of these four predictions. We have 14 African countries, 19 American countries, 33 Asian or Pacific countries, and 33 European countries in this network.

2. GDP. We use the GDP in 2014 as the second variable to predict. We label as 1 the countries with a GDP higher than the median; and otherwise 0.

3. Export complexity index (ECI). ECI measures the economic complexity for a country. For example, Japan has a high ECI because it exports products with high uniqueness. Similar to GDP, we label the countries with an ECI higher than the median as 1; and otherwise 0.

[2] https://aminer.org/socialtieacross

Description of Synthetic Dataset

As mentioned in the main article, we generate the network with two types of agents (e.g., buyers and sellers) who explore exchanges in their neighborhoods. We predict two individual characteristics in this dataset:

1. Type. As mentioned previously, we predict the type of each agent (a binary variable). The probability that the agent i is type A (e.g., buyers) is 0.5, and agents draw their types independently.

2. Location. We divide the 50×50 grid into four divisions: (1) $0 \leq x < 25$ and $0 \leq y < 25$; (2) $0 \leq x < 25$ and $25 \leq y < 50$; (3) $25 \leq x < 50$ and $0 \leq y < 25$; (4) $25 \leq x < 50$ and $25 \leq y < 50$. We predict whether a node belongs to each division, and then take the average AUC to measure the prediction performance for this characteristic.

Supplementary Note 3: Learning

Learning Method

Random noise. Since real-world social networks may incorporate random noises, we therefore add an *i.i.d.* random shock for the marginal utility into each pair (i, j). Following the conditions in Proposition (1), for $j \in S_i^*$,

$$\Delta u_i(j) + \epsilon_{ij} \geq 0; \tag{6}$$

and for $l \notin S_i^*$,

$$\min\Big(\Delta u_i(j), \Delta u_j(i)\Big) + \epsilon_{ij} < 0. \tag{7}$$

For computational simplicity, we assume that the CDF of random shock ϵ_{ij} is a sigmoid function,

$$\phi(x) = Pr[-\epsilon_{ij} \leq x] = \frac{1}{1 + \exp(-x - u_0)}. \tag{8}$$

We then establish the loss function. We learn the $\mathbf{W}$, $\mathbf{b}$, $\mathbf{c}$ by minimizing the loss function. u_0 can be considered as a baseline benefit or cost of forming a link. u_0 is

learned along with $\mathbf{b}$ and $\mathbf{c}$ (can be perceived as a reserved utility) but is omitted in later equations.

Loss. We next specify the definition of the loss function:

$$\mathcal{L} = \mathcal{L}_{\text{pos}} + \mathcal{L}_{\text{neg}} + \mathcal{L}_{\text{fp}} + \mathcal{L}_{\text{reg}}. \tag{9}$$

- $\mathcal{L}_{\text{pos}} = -\frac{\sum_{(i,j)\in\mathcal{E}} \log\left(\phi\left(|S_i^*|\Delta u_i(j)\right)\right)}{\sum_{(i,j)\in\mathcal{E}} 1}$. This loss measures how well connected pairs are predicted. $\phi(x)$ is the sigmoid function ($\phi(x) = \frac{1}{\exp(-x-u_0)+1}$). u_0 is an unknown scalar to be learned along with $\mathbf{b}$ and $\mathbf{c}$. Multiplying $|S_i^*|$ is to consider that fact that when an agent has many agents (a large $|S_i^*|$), the marginal benefit from each neighbor ($\Delta u_i(j)$) does not need to be very large. In other words, we require $\Delta u_i(j)$ to be large if i has few neighbors but not necessarily large if i has many neighbors.

- $\mathcal{L}_{\text{neg}} = -\frac{\sum_{(i,j)\notin\mathcal{E}} \log\left(1-\phi\left(\min\left(\Delta u_i(j), \Delta u_j(i)\right)\right)\right)}{\sum_{(i,j)\notin\mathcal{E}} 1}$. This loss measures how well disconnected pairs are predicted. This term follows the third condition in Proposition 1.

- $\mathcal{L}_{\text{fp}} = \lambda_{\text{fp}} \frac{\sum_{(i,j)\notin\text{FP}} \log\left(1-\phi\left(\min\left(\Delta u_i(j), \Delta u_j(i)\right)\right)\right)}{\sum_{(i,j)\in\mathcal{E}} 1}$. The penalty of existing false positives. FP is the set of pairs that are not in $\mathcal{E}$ but rank in the top $|\mathcal{E}|$ among all $\min(\Delta u_i(j), \Delta u_j(i)), \forall i, j$. Because the number of disconnected pairs is typically much larger than the number of connected pairs, false positives are usually not properly penalized. λ_{fp} scales the weight of this term.

- $\mathcal{L}_{\text{reg}} = \lambda_{\text{reg}}||\theta||_1$. Lasso ($\ell_1$) helps to eliminate unnecessary dimensions and to determine the choice of K. Therefore, if we learn a b_k or a c_k close to zero, we can remove this dimension and find an optimal K^*.

Dimensionality selection. We next provide a method to select the optimal dimensionality (K^*) along with (K^*_{bnf} and K^*_{cst}). Algorithm 1 presents the method. The philosophy is that for each K, we enumerate all possible K_{bnf}. "SGD" represents the algorithm for learning $\mathbf{W}$ and $\boldsymbol{\theta}$ via stochastic gradient descent and will be discussed later. If K_{bnf} gives rise to the best AUC for the current K, and the

corresponding $\boldsymbol{\theta}$ contains 0, then we terminate the enumeration. After removing the dimensions with zero-valued θ_k (< 0.01 in practice), we obtain the optimal dimensionality and the values of corresponding variables. We stop increasing K if we find a zero-valued θ_k in the optimal run in the best K_{bnf} for this K. A zero-valued θ_k means that the k-th dimension does not contribute to either benefits or costs so we can drop that dimension without impacting the performance of this model. We call such dimensions **"degenerate dimensions"**.

Result: $\mathbf{W}^*, \boldsymbol{\theta}^*, K^*, K^*_{\text{bnf}}, K^*_{\text{cst}}$

Initialize K to a small number;

repeat

  $K = K + 1$;

  $\mathcal{L}^*(K) = +\infty$;

  for $0 \leq K_{bnf} \leq K$ **do**

    $\mathbf{W}(K, K_{\text{bnf}}), \boldsymbol{\theta}(K, K_{\text{bnf}}) = \text{SGD}(\mathcal{L}, K, K_{\text{bnf}})$;

    Let $\mathcal{L}(K, K_{\text{bnf}})$ be the current loss;

    if $\mathcal{L}(K, K_{bnf}) < \mathcal{L}^*(K)$ **then**

      $\mathcal{L}^*(K) = \mathcal{L}(K, K_{\text{bnf}})$;

      $K^*_{\text{bnf}} = K_{\text{bnf}}$;

    end

  end

until $\exists k \ s.t. \ \theta_k \approx 0$;

$K^* = K - |\{\theta_k \approx 0\}|$;

$K^*_{\text{bnf}} = K^*_{\text{bnf}} - |\{b_k \approx 0\}|$;

$K^*_{\text{cst}} = K^* - K^*_{\text{bnf}}$;

$\mathbf{W}^* = \mathbf{W}(K^*, K^*_{\text{bnf}})$ (after removing the k-th dimension with $\boldsymbol{\theta}_k = 0$);

$\boldsymbol{\theta}^* = \boldsymbol{\theta}(K^*, K^*_{\text{bnf}})$;

Algorithm 1: The procedure of learning endowment vectors and other parameters.

Stochastic gradient descent (SGD) Here we describe the algorithm for SGD given K and K_{bnf}. We use TensorFlow (1) to implement the learning algorithm. Specifically, we use Adam optimizer (2) as the optimizer. We run a large number of iterations (5,000) to guarantee the convergence of learning. Importantly, because the memory cannot typically afford N^2 links as the input for one iteration, we

retain all the connected pairs as the input while randomly sampling the number of unconnected pairs. Specifically, we sample ten times the number of connected pairs each iteration (four times for Andorra because of the memory limit); however we still have a consistent estimation for $\mathcal{L}_{\text{neg}}$ in expectation. This technique is called "negative sampling" and is also used in many machine learning methods. For a typical network with a density of 0.1%, each iteration we sample approximately 1% pairs so every 100 iterations may cover all pairs on expectation. Also note that the stochastic nature of this learning process reduces the likelihood to converge to unwanted local minima. To approximate the optimum, we selected the run with the best AUC for link fitting.

Speed-up for Andorra Dataset. When the network is large (more than 10,000, e.g. Andorra) nodes, the computation of $\mathcal{L}_{\text{fp}}$ is very time-consuming. To speed up the learning, we could drop this term because empirically, this term is small for large-scale networks. For example, when learning endowment vectors for Andorra dataset, this term takes up less than 0.1% of the total loss even if the term is not optimized. Therefore, we drop $\mathcal{L}_{\text{fp}}$ in Andorra dataset to speed up the learning, but retain $\mathcal{L}_{\text{fp}}$ for other datasets to increase learning performance.

Choice of λ_{reg} and λ_{fp}. The value of λ_{reg} determines the constraint of the dimensionality and avoids overfitting. A large λ_{reg} will lead all b_k and c_k to be zero, while a zero-valued λ_{reg} fails to limit the dimensionality and the model may eventually overfit the data with an extremely high K. We select λ_{reg} *a priori* based on the complexity of the network. When the number of nodes is smaller than 1,000, we let $\lambda_{\text{reg}} = 0.1$; when the number of nodes is greater than 1,000 but smaller than 10,000, we set $\lambda_{\text{reg}} = 0.05$; otherwise we set $\lambda_{\text{reg}} = 0.01$. For λ_{fp}, we set it as 0.01 for all datasets. The good fitting and the prediction performance demonstrate that such selection is reasonable. Note that it is not necessary to obtain an optimal λ_{reg} or λ_{fp} if they result in satisfactory endowment vector estimations.

Learning rate and the number of iterations. There is no specific guidance to select these two terms *a priori*. Empirically, 0.1 is a reasonable initial learning rate, and it decays by 5% every 100 epochs. In addition, 5,000 can be a reasonable choice for the number of iterations. We will show in the next section that such choices result in sensible results.

Learning curves

Here we plot the learning curves for all datasets under the optimal settings. As described in *Methods*, we start from five random initial points to approximate the global optimum. As shown in Supplementary Figure 2 and Supplementary Figure 3, all runs converge during the first 1,000 iterations, meaning that our choices of the learning rate and the number of iterations are sensible. Moreover, most runs converge to similar losses and AUCs. This finding is important because it indicates that the local optimum is a good approximation for the global optimum.

Results of dimensionality selection

For each K, we have a $K^*_{\text{bnf}}(K)$ that results in the smallest loss. If a zero-valued b_k or c_k is learned at the setting of $K, K^*_{\text{bnf}}(K)$, we stop increasing K. Here we plot the losses for the best run for each pair of K, K_{bnf} for all datasets in Supplementary Figure 4 (except for Andorra) (we do not plot very small K for the convenience of visualization). The increment of K stopped at 4, 6, 5, 8, and 12 respectively for Karate, Trade, Synthetic, Movie and Company, respectively. When (K, K_{bnf}) is set to be $(4, 2)$, $(6, 3)$, $(5, 3)$, $(8, 5)$, and $(12, 4)$ for Karate, Trade, Synthetic, Movie and Company respectively, we derive the minimal losses. Then we drop degenerate dimensions: one costly dimension for Karate, Trade, Movie and Company; one beneficial dimension for synthetic. After deleting the degenerate dimensions, we derive (K^*, K^*_{bnf}): $(3, 2)$ for Karate, $(5, 3)$ for Trade, $(4, 2)$ for Synthetic, $(7, 4)$ for Movie, $(11, 4)$ for the Company dataset. For Andorra, because of the limit of computational resource, we are unable to enumerate for all (K, K_{bnf}). Instead, we set an arbitrary large $K = 16$ and reduce the dimensionality by the regularization term $\mathcal{L}_{\text{reg}}$, where we find one costly degenerate dimension. So we reduce one costly dimension, i.e. $K = 15$. Here we do not argue that 15 is an optimal choice for the Andorra dataset, but it is good enough for further agent-based modeling.

Supplementary Note 4: Graphical illustration

Illustration for the trade network

Since we have the trade network with only 100 countries, we can visualize the output of learned endowment vectors that is close to the optimal choice of dimensionality: $K = 6$ and $K_{\text{bnf}} = 4$. It is convenient for visualization to keep both K_{bnf} and K_{cst} even.

Supplementary Figure 5 depicts the distribution of endowment vectors for all countries in the network. The first four dimensions are beneficial dimensions (with $b_k > 0$). We find that China, United Arab Emirates, the US, and Germany are high in these four dimensions respectively. The last two dimensions are costly dimensions (with $c_k > 0$). We find that geographically close countries are clustered on the space, meaning that the distance between countries plays an important role in the costs of network formation.

Illustration for synthetic dataset

As mentioned in the main article, nodes are located on a 50×50 grid. Each node can be either a seller or a buyer (with probabilities of 0.5 independently). We plot the learned endowments in Supplementary Figure 6. The plots show that we have recovered the data generating process. In the left panel, red and blue nodes are randomly distributed, which is consistent with the fact that buyers and sellers are randomly distributed on the grid. In the right panel, we observe that the degree of blue increases along the x-axis and that the degree of red increases along the y-axis. When both x and y are small, black is shown; when both x and y are large, purple is shown. In sum, the right panel demonstrates that we have successfully recovered the data generation of costly dimensions.

Supplementary Note 5: Comparison with network embedding algorithms

Although we have emphasized that the proposed model is not intended for dimension reduction of graph structures, we compare it with a classical network embedding algorithm, DeepWalk. Our goal is not to invent algorithms to outperform DeepWalk or other network embedding algorithms. In addition, to remain interpretability (link formation mechanism) and model simplicity, we do not enforce similarity among nodes on randomly sampled paths, which is why DeepWalk and other network embedding algorithms sometimes perform better in node classification tasks than we do, especially when similar nodes are clustered on the networks. As shown in Supplementary Table 2, when the agents with this characteristic are densely clustered, the present model does not outperform DeepWalk, as expected. However, when characteristics do not show this "clustering" effect or may imply exchange effects (GDP in Trade, Type in Synthetic, Job in Movie), the present model *does* significantly outperform DeepWalk. This finding indicates that it is necessary to incorporate exchange effects along with coordination costs into network formation models, and that our model has a decent predictive power without losing interpretability in social science and link formation mechanisms.

Supplementary Note 6: "Social power or exclusion" for all networks

In the main article, we have presented the scatter plots about the correlation between social power or social exclusion and micro-level statistics of networks on the Andorra dataset. Here we show the correlations for other datasets in Supplementary Table 3. Because the synthetic network is a bipartite graph and the movie network is almost a bipartite graph (some agents served as both directors and cast members), clustering coefficients are inapplicable. We find consistent results for most datasets with the results for the Andorra dataset. For the company data, we do not observe the significant correlation between social power and degree; we conjecture that this is because members in high status (managers) of the company do not need

to communicate with every person.

Supplementary Note 7: Reconstruction of networks

Our methods to reconstruct the networks are as follows. Codes are provided online.

1. Learn the endowment vectors $\mathbf{W}$, and scaling parameters $\mathbf{b}$ and $\mathbf{c}$ with the aforementioned methods.

2. Rank the marginal utilities for all pairs (i, j) by equation (5) in main text (specifically, $\min(\Delta u_i(j), \Delta u_j(i))$) and establish a *reconstructed network* based on the top $|\mathcal{E}|$ pairs. Record the minimal utility $\min(\Delta u_i(j), \Delta u_j(i))$ in the reconstructed network, denoted by $\bar{u}$.

3. Multiple $\mathbf{c}$ by $1 - \alpha$, and repeat the previous step, to obtain *new reconstructed networks after decreasing* $\mathbf{c}$. In the reconstructed network, $\min(\Delta u_i(j), \Delta u_j(i)) \geq \bar{u}$.

4. Compute the statistics shown in Fig. (4) for each reconstructed network.

Supplementary Note 8: Robustness check: no splitting for beneficial and costly dimensions

We present the link fitting performance for the "split" (with beneficial and costly dimensions, as presented in the main article) and the "non-split" (b_k and c_k can be positive simultaneously) conditions. In Supplementary Table 4, with the same number of dimensions, the AUC of the "split" condition is better than the "non-split" condition for all datasets. Note that because the AUCs of link fitting are typically very high, a significant decrease in AUC indicates a worse modeling fitting ability.

We also use the learned endowment vectors on the "non-split" condition to predict individual characteristics. We present the comparison between the "split" and "non-split" conditions in Supplementary Table 5. We find that the prediction powers of endowments learned in the two conditions are comparable.

In addition, we also show that even if we do not force some b_k or c_k to be zero-valued in the "non-split" condition, we will still learn $\mathbf{b}^*$ and $\mathbf{c}^*$ such that for most k, either $b_k = 0$ or $c_k = 0$.

Supplementary Note 9: Robustness check: Variations of the functional form

We show the robustness of our model by using two variations of the functional form. The first one (*Abs*) is to change the G_i from ℓ_2 into an ℓ_1 norm:

$$\Delta u_i(j) = \sum_{k=1}^{K} b_k \max(w_{jk} - w_{ik}, 0) - \|\mathbf{c} \circ (\mathbf{w_j} - \mathbf{w_i})\|_1. \tag{10}$$

The second one (*Smooth*) is to use a smooth form of ReLU ($\max(x, 0)$) to assist the optimization:

$$\Delta u_i(j) = \sum_{k=1}^{K} b_k \log(1 + \exp(w_{jk} - w_{ik})) - \|\mathbf{c} \circ (\mathbf{w_j} - \mathbf{w_i})\|_2. \tag{11}$$

Note that when x is far away from 0, $\max(x, 0)$ is very close to $\log(1 + \exp(x))$. When x is around 0, $\log(1+\exp(x))$ has a smoother gradient than $\max(x, 0)$, which is more convenient for the optimization. We can also consider $\log(1 + \exp(x))$ as a "noisy" form of $\max(x, 0)$: when x is close to zero, a small random shock will influence whether there is a benefit.

To show that these two versions of functional forms will not influence the fitting ability and the predictive power of the learned endowments, we present the fitting performance in Supplementary Table 7 and the prediction performance in Supplementary Table 8. We find that the performances for three versions of utility forms yield very similar results; the similar results mean that in the main article, it is reasonable to only present one functional form.

Supplementary References

[1] Abadi, M. & TensorFlow, A. A. B. P. Large-scale machine learning on heterogeneous distributed systems. In *Proceedings of the 12th USENIX Symposium on Operating Systems Design and Implementation*, 265–283 (2016).

[2] Kingma, D. & Ba, J. Adam: a method for stochastic optimization. In *Proceedings of the 3rd International Conference on Learning Representations* (2015).
